# Addressing the overuse of hospital emergency departments in the Portuguese NHS: a new paradigm

**DOI:** 10.3389/fpubh.2024.1444951

**Published:** 2025-01-13

**Authors:** Francisco Goiana-da-Silva, Soraia Costa, Filipa Malcata, Juliana Sá, Rafael Vasconcelos, Miguel Cabral, Raisa Guedes, Inês Morais-Vilaça, Lara Pinheiro-Guedes, João Ferreira, Nélson Pereira, José Gaspar-Pais, Judite Neves, Joaquim Monteiro, Vera Pires, Miguel Paiva, Rui Guimarães, Hutan Ashrafian, Rita Moreira, Fátima Fonseca, Filomena Cardoso, Jaime Alves, Ara Darzi, Fernando Araújo

**Affiliations:** ^1^Centre for Health Policy, Institute of Global Health Innovation, Imperial College London, London, United Kingdom; ^2^NOVA Medical School, Universidade NOVA de Lisboa, Lisbon, Portugal; ^3^Faculdade de Ciências da Saúde, Universidade da Beira Interior, Covilhã, Portugal; ^4^Portuguese National Health Service Executive Board, Porto, Portugal; ^5^Public Health Unit, ULS Póvoa de Varzim/Vila do Conde, Póvoa de Varzim, Portugal; ^6^Public Health Unit, ULS São João, Porto, Portugal; ^7^Internal Medicine Department, ULS Santo António, Porto, Portugal; ^8^Public Health Unit, ULS Região de Leiria, Leiria, Portugal; ^9^Public Health Unit, ULS Gaia/Espinho, Vila Nova de Gaia, Portugal; ^10^Public Health Unit, ULS Tâmega e Sousa, Penafiel, Portugal; ^11^Management Board, ULS Lezíria, Santarém, Portugal; ^12^Management Board, ULS Tâmega e Sousa, Penafiel, Portugal; ^13^Management Board,ULS Póvoa de Varzim/Vila do Conde, Póvoa de Varzim, Portugal; ^14^Department of Family and Community Health, ULS Póvoa de Varzim/Vila do Conde, Póvoa de Varzim, Portugal; ^15^Management Board, ULS Entre Douro e Vouga, Santa Maria da Feira, Portugal; ^16^Management Board, ULS Gaia/Espinho, Vila Nova de Gaia, Portugal; ^17^Department of Surgery and Cancer, Faculty of Medicine, Imperial College London, London, United Kingdom; ^18^Escola Superior de Saúde, Universidade Fernando Pessoa, Porto, Portugal; ^19^Faculty of Medicine, University of Porto, Porto, Portugal

**Keywords:** emergency department, primary healthcare, triage, healthcare optimization, care integration

## Abstract

The escalating trend of inappropriate visits to Emergency Departments (ED) has led to significant concerns, including resource misallocation, compromised patient care, and an increased burden on healthcare workers. Portugal faces a notable challenge, reporting one of the highest *per capita* ED visit rates, with an annual average of approximately 6 million ED visits from 2013 to 2023. In response, the “Call First, Save Lives” pilot project was launched by the Portuguese NHS Executive Board, in 2023, at the Local Health Unit (LHU) of Póvoa de Varzim/Vila do Conde. This project leverages the SNS 24 telephone triage system to direct patients to the most appropriate care settings, alleviating pressure on ED, and optimizing healthcare resources. The project introduced several strategic enhancements, including optimizations of the SNS 24 system and inter-institutional initiatives to promote interoperability, hospital follow-ups for chronic conditions, and a “reverse referral” system directing non-urgent ED cases to Primary Care Services (PCS). In PCS, the project expanded acute care consultations and introduced complementary health services. The initiative also emphasized communication to improve health literacy and appropriate utilization of the NHS, supported by legal framework development. Since the beginning of the project, the number of calls received by SNS 24 showed an upward trend, both at the local, 27.6%, and national level, 44.5%, having already expanded to other LHU. Nonetheless, challenges such as the “reverse referral” process complexity, healthcare information systems integration, and procedural standardization need addressing, with future plans including satisfaction surveys and patient triage protocol refinements to improve service delivery and patient adherence.

## 1 Introduction

Emergency departments serve as multidisciplinary and multiprofessional services whose aim is to provide healthcare in all situations deemed clinically urgent or emergent. These terms typically describe acute clinical scenarios where the sudden compromise or failure of vital functions poses immediate risks, requiring prompt medical response (1).

As vital components of healthcare systems, EDs often represent the primary entry point for patients seeking care, offering immediate access without direct charges, which has inadvertently led to a notable misuse of services. Observations from various Organization for Economic Co-operation and Development (OECD) countries highlight a concerning trend: an escalating volume of ED visits, especially those categorized as “non-urgent” or “inappropriate” (2). Such visits encompass medical conditions that would be more suitably managed by Primary Care Services (PCS), indicating a misuse of emergency resources for issues that could be effectively addressed by general practitioners (GPs) or primary care teams (3).

The issue of “inappropriate” ED visits has garnered international attention for multiple reasons. A significant concern is the unnecessary consumption of ED resources (including healthcare professionals and medical equipment), which diverts attention from more critically ill patients and exacerbates the workload of healthcare providers (2, 4). These “non-urgent” visits compromise the ED’s capacity to deliver timely and safe care, reduces access to genuine emergencies, disrupt continuity of care, and contribute to burnout in healthcare professionals (5). Furthermore, they are associated with reduced patient satisfaction, diminished care quality, increased waiting times, delayed diagnoses, treatment delays, and potentially escalate the risk of violence toward healthcare workers (4, 6–9). In England, the financial toll of inappropriate ED visits was estimated to be nearly £100 million between 2011 and 2012 (3). In short, the use of the ED by patients with non-urgent medical conditions in itself implies a loss of efficiency for the services, compromising their performance and jeopardizing the satisfaction of professionals and users.

The scientific literature presents mixed evidence regarding interventions aimed at reducing avoidable ED visits, largely due to the variability in healthcare systems worldwide. Even though there is no consensus established, the studies tend to agree that reducing ED use will require a broad approach that integrates several interventions adapted to the country’s healthcare reality. Such reforms should include mechanisms for monitoring outcomes and unintended effects, with a rigorous evaluation. One of the most documented measures is the introduction of telephone triage systems, which, through the deployment of call centers staffed by clinically trained personnel and the integration with PCS, have shown promise in directing patients to appropriate care settings, thus potentially reducing inappropriate ED visits. These telephone triage systems are intended as a general means of entry to the health service and provider of health counseling, differing from Europe’s 112 emergency lines as they are not designed for emergency situations. The evidence also supports that increasing the supply of PCS is associated with lower ED visits and that, although difficult to standardize, educational interventions have the potential to reduce overall healthcare use (10–13).

Internationally, various strategies have been explored, with studies from Switzerland, Canada, Japan, Australia, Netherlands, and the United Kingdom documenting the positive impacts of telephone triage services, enhanced primary care access, and integrated care pathways on reducing unnecessary ED usage.

A randomized, cross-sectional investigation conducted in Switzerland revealed that most individuals utilizing telephone triage services adjusted their behaviors based on the guidance provided by nurses. In the absence of access to these services, a significant portion of these individuals would likely have sought care at ED. This access was associated with an estimated 28% reduction in the likelihood of intending to visit the ED (14). Similarly, a study in Ontario, Canada, examining telephone triage advice reported a decrease in the inclination of callers to visit the ED (15).

An observational study in Australia, focusing on a cohort of adults aged 45 and above who utilized the national telephone triage system, demonstrated high compliance rates, noting that only 7% of these interactions leading to self-referral to the ED within 24 h with higher incidence observed among individuals from disadvantaged communities, calls placed after standard hours, or calls initiated on behalf of the patient, especially when the initial intention was to visit the ED (11, 16).

In the Netherlands, the enhancement of primary care access afterhours through the establishment of “emergency care access points” as primary care physician cooperatives, resulted in a 13 to 22% reduction in ED utilization, proving to be both safe and cost-effective (17). Moreover, in the United Kingdom, the practice of redirecting patients, who were evaluated on-scene by paramedics, to General Practitioners based on urgency assessments, demonstrated a significant capacity to safely reduce unnecessary ED transfers, accurately identify the most appropriate care setting for each patient, and improve emergency care pathways at a systemic level (18).

Regarding Portugal, the scenario is particularly striking, with the highest *per capita* ED visit rate among OECD countries in 2011, noting over 70 visits per 100 population, which contrasts with the mean of 31 per 100 population of the region (2). Accordingly, to data from the *Portal da Transparência*,^1^ an open online source with data on all ED of public hospitals from mainland Portugal, there was an annual average of approximately 6 million ED visits between 2013 and 2023, equating to 58 visits per 100 population. Within the period in analysis, there has been a significant decrease in ED attendance in 2020 (nearly 32.8%) due to the COVID-19 pandemic, with a consistent increase in the following years (2021: 9.3%; 2022: 13.3%), returning to the basal levels registered before the pandemic (19).

In Portugal, the Triage of Manchester (TM) system is employed to stratify the urgency of observation for every patient upon arrival at the ED. Regarding the classification of emergency episodes according to TM, the period from 2003 to 2023 exhibited a stable distribution, with emergent cases (“color red”—requiring immediate care) constituting an average of 0.4% of visits; very urgent cases (“color orange”—with a maximum 10-min wait for observation) accounting for approximately 11% of episodes; urgent cases (“color yellow”—with a maximum 60-min wait for observation) representing about 47% of all episodes; standard (“color green”—permitting up to 120 min for observation) and non-urgent cases (“color blue”—allowing up to 240 min for observation) comprising 37 and 2%, respectively, of ED visits. Another category, denoted by the color “white,” encompasses situations where there is an incorrect utilization (for non-necessity) of the ED, constituting approximately 3% of cases. Throughout the study period, the proportions of each TM classification remained consistent, providing insights into the distribution of urgency levels among patients seeking care at the ED in Portugal (20).

Also, in Portugal, there is already a telephone triage system for health advice and guidance of users in the National Health Service, called SNS 24, which differs from the 112—emergency telephone number which is dedicated to emergencies—operated by National Institute of Medical Emergency (INEM) and its partners.

Opting for a family physician consultation instead of an emergency room visit might lead to significant cost savings. If we consider the lowest (21.63€) and highest (38.04€) hourly wage for the most common contract of family doctors (*Dedicação Plena*) (21) and the price (85.91€) associated with an admission to a regular ED (*Serviço de Urgência Médico-Cirúrgica*) (22) the potential savings become clear. Simple estimations, without taking into account additional costs (diagnostic tests, for example) or externalities, show us that: for a 15-min consultation the savings range from €80.50 (based on the lowest hourly wage) to €76.40 (based on the highest hourly wage); for a 30-min consultation, the savings range from €75.10 to €66.89, respectively; in the case of a 60-min consultation, the savings range from €64.28 to €47.87. Thus, there was a need to create solutions to the problem of over utilization and inadequate use of ED in Portugal, ideally by promoting appropriate navigation through the National Health Service necessarily maintaining or improving the standards of quality and access that have been established until now. It is in the context of this identified health need that this project “Call First, Save Lifes” was developed.

## 2 Context (setting and population)

Following a comprehensive assessment across the mainland territory, Póvoa de Varzim and Vila do Conde, located in the Northern region of Portugal, were selected as the implementation sites for this pilot project. These municipalities were chosen based on their geographical and demographic attributes, with a notably compact geographical layout (around 231 km2), which facilitates quick access to remote areas. The combined population of approximately 150,000 is supported by robust and nationally recognized health services infrastructure. In 2022, this region achieved the highest national score in the Global Performance Index (GPI) for PCS with nearly all patients having a local primary care service -Family Health Unit type B where all health professionals have a GPI and financial incentives, and being assigned to a family doctor, ensuring comprehensive healthcare coverage and response to healthcare needs (23).

Hospital care in these municipalities is delivered through a hospital center encompassing two district hospitals equipped with various facilities, including an efficiently organized ED. The percentage of “avoidable” ED visits of the local hospital center follows the national tendency, with the episodes screened with “Green,” “Blue” and “White” representing almost 50% of all episodes in 2022 (21).

Since January 2024 the Hospital and PCS where integrated into the same health services structure, the Local Health Unit (LHU) of Póvoa de Varzim/Vila do Conde, an organizational model transition prepared since 2023.

The region was also well-served by the comprehensive coverage and effective functioning of the Portuguese Integrated Medical Emergency System. This system ensures the availability of pre-hospital care for individuals in emergency scenarios. Furthermore, the contribution of local authorities was pivotal, attributed to their dynamic and exemplary support for healthcare services, reinforcing the framework for emergency medical care delivery (23).

## 3 Detail to understand key programmatic elements

The pilot project “Call First, Save Lives” (Portuguese title: “Ligue Antes, Salve Vidas”) was a pioneering project initiated to encourage individuals to utilize SNS 24 aiming to ensure the provision of timely and appropriate healthcare for all patients, alleviate excessive demand on emergency care services through proper patient referral, and decrease the unwarranted utilization of primary and hospital healthcare services, thereby enhancing health outcomes. The project was conceptualized and managed by the Executive Board of the Portuguese National Health Service (DE-SNS), in collaboration with the Shared Services of the Ministry of Health (SPMS), an entity that offers logistical, financial and human resources, information and communication systems, and technologies support to healthcare organizations, alongside local government partners (24).

The project was executed in various phases, with the inaugural phase (First Phase) spanning from 24 May 2023 to 15th January 2024 and the Second Phase from 16th January 2024 until present time (24).

The First Phase of the project focused on laying the strategic foundations, centered around two core strategies for its entire development: (a) enhancing the healthcare service’s efficiency and organization, and (b) launching educational campaigns and training initiatives to elevate health literacy among the population. The measures implemented and the organizations directly involved in each are described in Table 1.

**Table 1:** Measures implemented during the first phase of the project in LHU Póvoa de Varzim/Vila do Conde, according to strategic axis and directly involved institutions.

Strategy	Institution	Measures
Enhancing the healthcare service’s efficiency and organization	SNS24	Direct referral of patients to the most suitable care setting, enabling direct appointment scheduling within PCS
		Implementation of a system for remotely issuing self-certified short-term sick leave declarations
		Continuous refinement of triage algorithms
	PCS	Increased availability of acute care consultations in all care units
		Launching of a complementary primary health service (“Serviço de Atendimento Complementar”) on weekends and public holidays to maintain the supply of primary healthcare for non-urgent medical conditions
		Optimization of the home hospitalization protocol for acutely ill patients (integration with hospital services)
	Hospital Care	Improved response to follow-up patients with chronic pathology through PCS referral and with guaranteed response within 48 h
		Introduced post-ED visit reassessment consultations across clinical specialties
		Termination of the episodes categorized as “white” in MT system by optimizing the functioning and referencing of hospital services
		Implementation of a “reverse referral” system from the ED to PCS for patients deemed least urgent (“color green”) or non-urgent (“color blue”), according to a normative circular in force from the Central Administration of the Health System
		Provision of sick leave documentation directly in the ED
		Optimization of the home hospitalization protocol for acutely ill patients (integration with PCS)
	INEM	Improvements to the algorithms
	SPMS	Upgrading system interoperability between the different levels of care
		Creation of data analysis and visualization tools for monitoring and evaluation
Communication and Education Strategies	DE-SNS, SPMS, Municipalities, LHU Póvoa de Varzim/Vila do Conde	Deployment of local and national media campaigns—leveraging local influencers to enhance health literacy, emphasizing self-care and guiding appropriate NHS utilization
	LHU Póvoa de Varzim/Vila do Conde, INEM, Municipalities	Training programs covering PCS, hospital services, and community engagement, including basic life support for healthcare professionals

One of the measures at the center of the project was the implementation of a “reverse referral” protocol in the ED, which made possible to refer patients considered to be of little or no urgency from the ED to the PCS. This allowed an alternative route for these patients and, through association with the other measures implemented, maintained the standards of quality of care and access that had been established until then. These measures include the ability of SNS 24 to directly book appointments on the system used by primary healthcare, something that was not previously available, as well as the ability to remotely issue a self-certified sick leave declaration to users who need to maintain self-care, avoiding the hitherto established procedure of having to go to their attending doctor in their family health unit to provide the declaration. Also, measures focussed on continuous access to primary healthcare provision for acute illness were prioritized.

In this First Phase, the reverse referral protocol was only applied in the general ED of the LHU hospital services, for people aged 18 and over, while all the other measures were aimed at all the resident population of the two municipalities, Póvoa de Varzim and Vila do Conde.

The Second Phase was implemented based on the creation of new legislation that provided for patients with green or blue color in Triage of Manchester, who were not referred or excluded from this project (due to medical reasons), to go to PCS, with a scheduled consultation within the first 24 h, instead of Hospital care. This legal update provided for compulsory referral if the defined criteria were met, differing from the previous framework which made it optional for patients to be referred. This framework ensured that the patients always received a response within the NHS at the appropriate location.

While in this Second Phase this procedure was applied only in the general ED, the Third Phase is planned to start in April 2024 expanding the project to the pediatric population aged one and over, in the LHU pediatric ED.

The regulatory frameworks were established to support the project’s implementation, with specific exceptions outlined for the “reverse referral” process within hospital ED. These exemptions, ensuring comprehensive and accessible emergency care for all patient categories, included: patients transported to the ED by ambulance, after indication from Urgent Patient Guidance Centers or INEM; patients referred by SNS 24; patients referred by PCS; patients referred by a doctor; patients bedridden or in a wheelchair, without the possibility of mobilization by their own means; patients aged 70 or over; patients victims of trauma; patients with acute psychiatric gynecological/obstetric or pediatric situations; patients accompanied by security forces; patients with indication of forensic medical expertise; and patients that follow specific screening flowcharts with severity and need for immediate action associated (25).

## 4 Results

**Figure 1:**
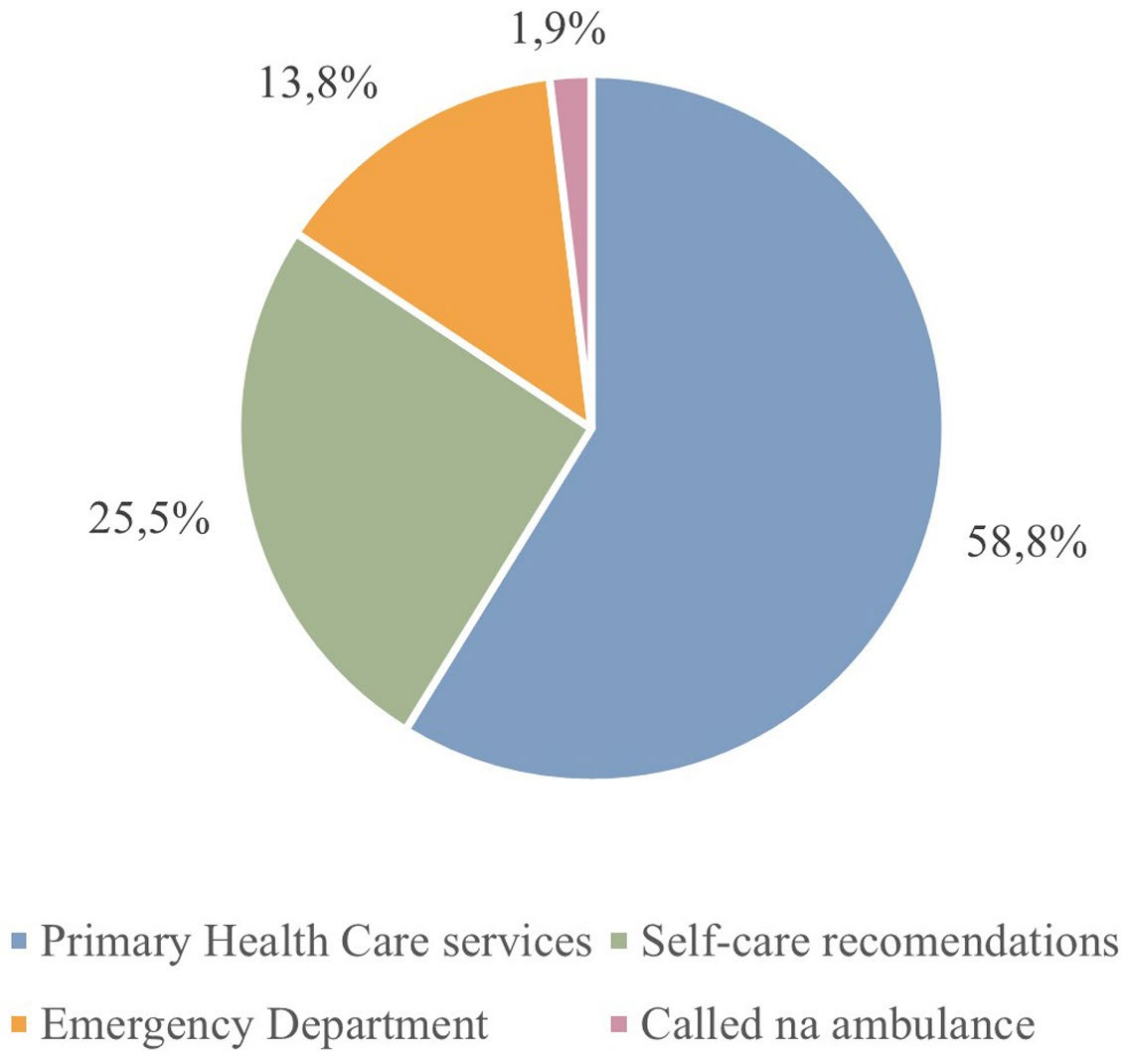
Percentage of referrals via SNS 24 in Póvoa de Varzim/Vila do Conde during first phase of the pilot project implementation.

Concerning the results of the First Phase of implementation:

- 51537 telephone triages were made to SNS 24, and 25908 patients (50.3%) were eligible for this project, meaning that their contact was related with an acute non-urgent medical condition (Figure 1).
- A total of 18179 appointments in primary care were successfully scheduled, achieving a scheduling success rate of 70%.
- Regarding the training and educational initiatives in PCS, 333 primary care professionals received training in basic life support with automatic external defibrillation, representing 90.2% of all primary care professionals.
- Regarding admissions to the ED by origin, since the start of the project the percentage of non-referred episodes in the general ED has fallen by 16 percentage points (from 58.7% to 43.2%). This reduction in non-referred situations is directly related to the increase in situations referred to by SNS 24, which rose from around 9 to 25%.
- Even though the reverse protocol was not implemented in the pediatric ED, the admissions of non-referred episodes to this ED have fallen by 14 percentage points (from 87.9% to 74.0%) and the referrals by SNS 24 rose from 7 to 20%.

Regarding the Second Phase of implementation of this Project, Table 2 shows the percentages of ED users not referred to the LHU who belong to the same complexity group as the Póvoa de Varzim/Vila do Conde LHU (2023 organizational model), for comparison purposes (26).

**Table 2:** Evolution of the percentage of non-referred episodes, before and during first and second phases of implementation of this project, in the LHU with hospitals in the same complexity group (group B).

	Before implementation	First phase	Second phase
LHU Póvoa de Varzim/Vila do Conde	70.20%	56.48%	30.96%
LHU Baixo Mondego	79.02%	78.33%	72.75%
LHU Barcelos/Esposende	72.58%	71.69%	66.60%
LHU Castelo Branco	71.87%	71.23%	68.16%
LHU Litoral Alentejano	81.32%	80.73%	80.50%
LHU Médio Ave	71.56%	70.82%	64.83%
LHU Nordeste	75.91%	76.80%	74.78%
ULS Guarda	71.63%	72.88%	70.91%

From the beginning to the current period of the project’s implementation, the number of calls received by SNS 24 showed an upward trend, both at the LHU Póvoa de Varzim/Vila do Conde level, with a 27.58% increase, and at the national level, with a 44.49% increase. Given the success of the pilot project at the LHU PVVC, it was expanded to the LHU of Entre Douro e Vouga and Gaia/Espinho, which encompasses about 1 million of citizens, about 10% of the Portuguese population.

Preparation for this expansion began in the end of 2023 and the terms of its implementation have been laid down in separate legislation. The project officially began, in both institutions, on the 5^th^ of March (24).

In Gaia/Espinho the ED had, in 2023, an average of 540 daily visits, 31% of which were referred. In its General ED, episodes triaged as “green” or “blue” accounted for 26% of total attendances, 80% of these without referral. During the first weeks of the project, there was a downward trend in the number of non-referred episodes compared to the previous year. By the end of March, the percentage of referrals to the General ED had risen from 37.7 to 70%. In the Pediatrics ED, the percentage of cases referred increased from 20 to 67%. It is important to note that the LHU Gaia/Espinho had already implemented a reverse referral protocol and that one of the difficulties encountered in this procedure was the lack of integration of PCS, which this project has remedied by optimizing the integration of the different levels of service.

In the ED of Entre Douro e Vouga, during 2023, in the General ER the episodes triaged with “green” accounted for 17.9%, “blue” for 1.8% and “white” for 2%. In the Pediatric ER, cases triaged with “green” accounted for 73.8% of the total, with “blue” accounting for 0.2% and “white” for 1.4%. Regarding the implementation of the project, in the first few weeks there was a 53-percentage point reduction in non-referred episodes in the ED services.

Due to these impressive results, the plan built by the DE-SNS predicted the extension of this emblematic project, evolving the professionals, institutions, civil communities and citizens, gradually and sequentially, to all territory of the continental country, during the year of 2024.

## 5 Discussion

In the Strengths, Weaknesses, Opportunities, and Threats (SWOT) analysis of this pilot project, several significant strengths and opportunities were identified, underscoring the initiative’s robust foundation and potential for success. A notable strength was the active engagement and commitment of the healthcare and management teams across all participating institutions. This commitment was pivotal in fostering a collaborative environment conducive to the project’s objectives. PCS were well-equipped with the necessary infrastructure and resources to augment their teams, accommodating the influx of patients, a crucial aspect for managing increased patient volumes efficiently. Furthermore, PCS demonstrated remarkable flexibility in adjusting the availability of acute care consultations, facilitating enhancements in the internal organization, with dedicated professionals efficiently addressing acute care needs.

Another key strength was the adaptability of the SNS 24 in aligning its schedules with the fluctuating resources and demand, ensuring an optimal balance between service provision and patient needs, contributing to the efficient utilization of healthcare resources, maximizing the impact of the triage system.

Another important aspect was the fact that it was placed a “yellow phone” in the ED that was directly connected to SNS24, to facilitate, guide, and simultaneously promote the culture of calling first.

Moreover, the project fostered a culture of continuous improvement, supported by rigorous feedback systems, enabling the timely identification and rectification of any weaknesses or failures. This proactive approach to quality improvement was instrumental in enhancing the effectiveness and efficiency of the pilot project, laying a solid foundation for future scalability and replication.

## 6 Acknowledgment of constraints

Regarding the Weaknesses and Threats identified, one notable challenge is the extensive list of exceptions related to the “reverse referral” process. According to emergency department professionals, it complicates performance and hinders standardization, due to the broad scope of clinical situations that end up being included by the exceptions, which, in non-severe situations or delayed search of care, would not justify an exception.

The optimal integration of healthcare information systems, firstly between primary care and hospital care, but also with other institutions involved, is still difficult to achieve, considering the initial complexity of these systems. There is also a need to revise the clinical algorithms to enhance the efficiency of the SNS 24 response and the accuracy of the triage algorithm itself. Even after the telephone triage service, almost half of the patients directed to an ED visit are still classified as standard cases (“color green”) and non-urgent cases (“color blue”) in the TM classification. Moreover, the flow of information among the various entities involved, not only those directly engaged in the project but also those in supporting roles, can be problematic. This issue may lead to delays in the execution of improvements.

Regarding reporting limitations, since most of the data was previously processed by the institutions themselves, there is a risk of information bias.

Furthermore, given the pilot nature of the project, the selection of the institutions was based on their good results in previous evaluations at national level, so that the project could be implemented with prosperity and in an environment suitable for easy adaptation and readjustment of measures. Therefore, we cannot exclude the selection bias in the obtained results.

To address some of these challenges and improve the project based on feedback, the plan includes conducting satisfaction surveys among both patients and hospital care professionals involved. This initiative aims to better understand and meet the needs identified through this evaluation.

The authors consider that the inter-institutional collaboration of local, national and international levels, in the production of this article is an added value for the quality of the information presented and bias control.

## 7 Conclusion

Both the first and the second phase of the “Call first, save lives” initiative showed robust, consistent and very promising results.

Understanding the profiles of patients who are less inclined to adhere to telephone triage recommendations, as well as identifying call characteristics that may impact compliance, will aid in enhance patient triage protocols, refine referral processes, inform staff training, and customize service design and delivery to maximize patient adherence.

As far as the next steps are concerned, it will be crucial to maintain the ongoing monitoring process, adapting it to the changes to be implemented. In addition, it will be relevant to study the referral algorithms used by the SNS 24 line in order to optimize the use of EDs for urgent situations. From this perspective, it is important to understand how the referrals made by the different actors have impacted patients’ health status in the medium/long term and to integrate the contributions of all stakeholders.

Building on all the experience gathered along the first and second phases of this initiative, the “Call first, save lives” project is already a ground-breaking innovation in the ED management and human resources allocation efficiency. Therefore, building on all the learning gathered, and taking into consideration necessary adaptations essential processes to specific geographical contexts, to be made sequentially, the potential strategic gains from the expansion of this project nationwide are clear. Therefore, such opportunity shall be taken into consideration by decision makers, as the principles of evidence-based policies aim for.

Furthermore, considering that the problem of overcrowding and inappropriate use of EDs is an international one, this project could be an example of good practice, and its procedures and results could serve as a starting point for implementing similar projects in other countries, without neglecting the process of adaptation necessary for each reality and health service. This is especially important in the European context, where health systems are more similar in nature, particularly in those with a health system similar to Portugal’s, such as Spain’s National Health System or the United Kingdom’s, and where ED overcrowding is a health need prioritized for intervention. Furthermore, replicating this project in other countries would be interesting to gage its reproducibility in other contexts.

## Data Availability

The raw data supporting the conclusions of this article will be made available by the authors, without undue reservation.

## Footnotes

^1^ https://www.sns.gov.pt/transparencia/
