# Correction: Addressing the overuse of hospital emergency departments in the Portuguese NHS: a new paradigm

**DOI:** 10.3389/fpubh.2025.1628755

**Published:** 2025-09-25

**Authors:** Francisco Goiana-da-Silva, Soraia Costa, Filipa Malcata, Juliana Sá, Rafael Vasconcelos, Miguel Cabral, Raisa Guedes, Inês Morais-Vilaça, Lara Pinheiro-Guedes, João Ferreira, Nélson Pereira, José Gaspar-Pais, Judite Neves, Joaquim Monteiro, Vera Pires, Miguel Paiva, Rui Guimarães, Hutan Ashrafian, Rita Moreira, Fátima Fonseca, Filomena Cardoso, Jaime Alves, Ara Darzi, Fernando Araújo

**Affiliations:** ^1^Centre for Health Policy, Institute of Global Health Innovation, Imperial College London, London, United Kingdom; ^2^NOVA Medical School, Universidade NOVA de Lisboa, Lisbon, Portugal; ^3^Faculdade de Ciências da Saúde, Universidade da Beira Interior, Covilhã, Portugal; ^4^Portuguese National Health Service Executive Board, Porto, Portugal; ^5^Public Health Unit, ULS Póvoa de Varzim/Vila do Conde, Póvoa de Varzim, Portugal; ^6^Public Health Unit, ULS São João, Porto, Portugal; ^7^Internal Medicine Department, ULS Santo António, Porto, Portugal; ^8^Public Health Unit, ULS Região de Leiria, Leiria, Portugal; ^9^Public Health Unit, ULS Gaia/Espinho, Vila Nova de Gaia, Portugal; ^10^Public Health Unit, ULS Tâmega e Sousa, Penafiel, Portugal; ^11^Management Board, ULS Lezíria, Santarém, Portugal; ^12^Management Board, ULS Tâmega e Sousa, Penafiel, Portugal; ^13^Management Board, ULS Póvoa de Varzim/Vila do Conde, Póvoa de Varzim, Portugal; ^14^Department of Family and Community Health, ULS Póvoa de Varzim/Vila do Conde, Póvoa de Varzim, Portugal; ^15^Management Board, ULS Entre Douro e Vouga, Santa Maria da Feira, Portugal; ^16^Management Board, ULS Gaia/Espinho, Vila Nova de Gaia, Portugal; ^17^Department of Surgery and Cancer, Faculty of Medicine, Imperial College London, London, United Kingdom; ^18^Escola Superior de Saúde, Universidade Fernando Pessoa, Porto, Portugal; ^19^Faculty of Medicine, University of Porto, Porto, Portugal

**Keywords:** emergency department, primary healthcare, triage, healthcare optimization, care integration

In the published article, author Juliana Sá was erroneously assigned as corresponding author. The correct corresponding author is Soraia Costa.

In the published article, the reference for “Furthermore, the contribution of local authorities was pivotal, attributed to their dynamic and exemplary support for healthcare services, reinforcing the framework for emergency medical care delivery” was incorrectly written as (21) Sindicato dos Médicos do Norte. Tabela Salarial (2024). Available at: https://www.sindicatomedicosnorte.pt/conteudos/10107/tabela-salarial-2024/ [Accessed October 21, 2024].

It should be (23) Ministério da Saúde. *Portaria n*.° *438/2023, de 15 de dezembro, do Ministério da Saúde. Diário da República n*.° *241/2023, Série I de 2023-12-15 Portugal*. (2023). p. 46–49.

In the published article, the reference for “The project was conceptualized and managed by the Executive Board of the Portuguese National Health Service (DE-SNS), in collaboration with the Shared Services of the Ministry of Health (SPMS), an entity that offers logistical, financial and human resources, information and communication systems, and technologies support to healthcare organizations, alongside local government partners.” was incorrectly written as (22) República Portuguesa. Portaria n.° 207/2017 | DR. Diário da República n.° 132/2017, Série I de 2017-07-11, Portugal; (2017). Available at: https://diariodarepublica.pt/dr/detalhe/portaria/207-2017-10766915723. Ministério da Saúde. Portaria n.° 438/202.

It should be (24) Direção Executiva do Serviço Nacional de Saúde. *Apresentação do projeto-piloto “Ligue antes, salve vidas” Área de abrangência do ACeS Póvoa do Varzim/Vila do Conde*. (2024).

In the published article, there was an error. There was a description that was unintentionally deleted during the revision process and an error in the numbers presented, and respective description, in the paragraph referring to the costs saved from the intervention.

A correction has been made to **Introduction**, Paragraphs 11 and 12. These sentences previously stated:

“Also, in Portugal, there is already a telephone triage system for health advice and guidance of users in the National Health Service, called SNS 24. The latter is dedicated to emergencies and operated by the National Institute of Medical Emergency (INEM) and its partners.

Opting for a family physician consultation instead of an emergency room visit might lead to significant cost savings. Simple estimations, without taking into account additional costs (diagnostic tests, for example) or externalities, show us that: for a 15-min consultation the savings range from €80.57 (based on the lowest hourly wage) to €76.40 (based on the highest hourly wage); for a 30-min consultation, the savings range from €75.23 to €66.89, respectively; in the case of a 60-min consultation, the savings increase further, ranging from €64.55 to €47.87 (21, 22). Thus, there was a need to create solutions to the problem of over-utilization and inadequate use of ED in Portugal, ideally by promoting appropriate navigation through the national health service necessarily maintaining or improving the standards of quality and access that have been established until now. It is in the context of this identified health need that this project “Call First, Save Lifes” was developed.”

The corrected sentence appears below:

“Also, in Portugal, there is already a telephone triage system for health advice and guidance of users in the National Health Service, called SNS 24, which differs from the 112—emergency telephone number which is dedicated to emergencies—operated by National Institute of Medical Emergency (INEM) and its partners.

Opting for a family physician consultation instead of an emergency room visit might lead to significant cost savings. If we consider the lowest (21.63€) and highest (38.04€) hourly wage for the most common contract of family doctors (*Dedicação Plena*) (21) and the price (85.91€) associated with an admission to a regular ED (*Serviço de Urgência Médico-Cirúrgica*) (22) the potential savings become clear. Simple estimations, without taking into account additional costs (diagnostic tests, for example) or externalities, show us that: for a 15-min consultation the savings range from €80.50 (based on the lowest hourly wage) to €76.40 (based on the highest hourly wage); for a 30-min consultation, the savings range from €75.10 to €66.89, respectively; in the case of a 60-min consultation, the savings range from €64.28 to €47.87. Thus, there was a need to create solutions to the problem of over-utilization and inadequate use of ED in Portugal, ideally by promoting appropriate navigation through the National Health Service necessarily maintaining or improving the standards of quality and access that have been established until now. It is in the context of this identified health need that this project “Call First, Save Lifes” was developed.”

The original article has been updated.
